# Health Disparities Past the Classroom: The Impact of Frontier Medicine on Students

**DOI:** 10.7759/cureus.6796

**Published:** 2020-01-28

**Authors:** Meghan White, Norma Perez, Sema Hajmurad, Melissa Victory

**Affiliations:** 1 Frontera de Salud, University of Texas Medical Branch, Galveston, USA; 2 Office of Student Affairs and Admissions, University of Texas Medical Branch, Galveston, USA

**Keywords:** study-away programs, health disparities, medically underserved communities, student perspective/impact

## Abstract

In 2000, the Liaison Committee on Medical Education issued a standard for cultural competence for medical students, stating the necessity of understanding different belief systems and cultures and how biases can affect health care and perpetuate health disparities. While many programs travel overseas to address this standard, our study evaluated an in-state, study-away, service-learning mission trip program’s efficacy of improving cultural competence and awareness of health disparities, as well as facilitating the ease of participation for students. Our overall goal was to provide a rich field opportunity in our own backyard that would allow students to visit a foreign environment without leaving the country, simultaneously eliminating the financial burden that comes with international travel and to expose the students to health disparities through partnership with local community centers and clinics present in the frontier region of southwest Texas (2016 - 2018). Post-trip assessments revealed that students were surprised by the disparities found within the state and gained a better understanding of community need. Subsequently, students revealed the desire to pursue careers that administer care to underserved populations and to improve their Spanish language skills. We concluded that this program increased awareness of cultural competency comparable to study-abroad programs. Post-trip evaluations were helpful in assessing change and impact on the students' cultural and health disparities awareness.

## Introduction

The Liaison Committee on Medical Education (LCME) upholds the importance of cultural competency training among medical students in order to ameliorate health disparities seen in the United States (US). The LCME affirms that the medical school curriculum should include “the recognition and development of solutions for healthcare disparities and the importance of meeting the healthcare needs of medically underserved populations.” Medical schools should also offer “sufficient opportunities for (encouragement of) and support (for) medical student participation in service-learning and community service activities [1].” The integration of these principles can be difficult to achieve within the walls of a classroom. As such, many “study abroad” programs have arisen to permit students to travel to international locations to learn these skills. Alternatively, there are “study-away” programs that can provide similar opportunities to students with less financial and time commitment burden. Study-away is described as “the concept and educational strategy that integrates study abroad programs with domestic programs" [2]. We use this term to stress that our program promotes the same goals of study-abroad, such as cultural competency development but is achieved by sending students to a domestic and yet unfamiliar location. Considering this, the importance of study-away and service-learning programs will be discussed to set the background that justifies mission trips to southwest Texas for students in healthcare professional programs. The mission trip highlights local health disparities and demonstrates the impact on students of service-learning experiences within study-away programs. The purpose of our evaluation is to determine if the aims of improved cultural competence and awareness of health disparities are achieved with such “study-away” programs.

Study-away and service-learning programs

International medical mission trips have become popular among students applying to and currently in healthcare professional programs. These trips often require extensive fundraising for travel outside of the US to administer care to communities in dire need. Thus, students can be limited by the financial cost. Instead, the development of study-away programs can promote similar opportunities at a lower financial burden. Even programs that have originated in border states and visit towns in Mexico along the US-Mexico border experienced international difficulties due to transportation limitations, passport difficulties, and delays at Border Customs [3]. Implementing programs that stay stateside reduces the barriers associated with foreign travel. A recent study reported that major factors contributing to students’ hesitancy to participate in service-learning study-abroad opportunities were “transport times and costs, social isolation, and access to resources and administrative tasks" [4]. Creating opportunities for students to experience study-away programs closer to the home could alleviate some of these concerns and increase student involvement in this type of service-learning.

Aside from the low cost and being close to home, study-away programs have been shown to equally achieve the goals of study-abroad programs: immersion and comfortability in a different community close to home or your own backyard. Each state encompasses a range of diverse cultures, religions, levels of health literacy, and community differences. Health profession students can find themselves in a community that vastly differs from their normal surroundings just by driving a bit away from their well-developed medical center and school. At this small distance, students have the chance to develop the same skill set as those who travel 3,000 miles. With study-away programs, students gain the same competencies as with global travel, but with minimal cost and travel time, while maximizing awareness of the health disparities within the US [2].

These study-away programs provide the opportunity for students to become more integrated with the community they are serving. By integrating study-away programs with ongoing community interventions, students develop a greater understanding of the community’s health needs and resources. Furthermore, they are more likely to report feeling that they have made a positive impact on the community, which increases their future participation in community service activities [5]. An added benefit of these opportunities is the development of leadership skills that help students become community leaders and “strong advocates for health equity" [6]. Thus, students who participate in these hands-on clinical learning experiences report that they are more likely to practice medicine in underserved areas compared to students who lack this exposure [7].

Service-learning programs provide a myriad of benefits for medical students that cannot be effectively taught in traditional classroom settings. In addition to being exposed to diverse and unequal healthcare in underserved communities, students expand their professional development by improving clinical and problem-solving skills and increasing their public health understanding. Students expand their personal development with improved communication, teamwork, and leadership skills. Furthermore, students enhance their senses of civic belonging and social justice by learning the cycle of healthcare problems that plague underserved communities within their home country. Students are challenged to consider how societal rules, politics, and economics play into the health of the patients seen in these rural settings in the US [8]. Cooperating with local programs ensures that students join the local community in a joint effort instead of providing care as a charitable act [6]. Joining community partners provides the appropriate guidance and encouragement for students to foster cultural humility and observe how local patients use the community's resources to determine their healthcare. This community partnership also ensures that these communities benefit from the study-away program [9]. As a result, students develop their social responsibility to motivate the community to further understand and support their own needs and leverage assets [10].

The idealism of students pursuing healthcare careers can be rejuvenated by interacting with community members fighting for the same end goals: health and wellness. The partnership creates empathy and understanding [11]. The despair and burnout commonly seen in the medical field can be combatted with a civic duty to promote healthcare outside of a hospital or clinic [10]. Engaging students in service-learning programs in distant, but still domestic, communities will impact their professional goals; after such experiences, many students have a newfound passion to work in rural or underserved locations [5, 7, 12]

Inspired by the benefits of study-away and service-learning programs, a student-directed, faculty-guided organization called Frontera de Salud (FdS), through the University of Texas Medical Branch (UTMB) at Galveston, TX, has developed a program to expose students to frontier and rural medicine. Visiting Presidio County, TX provides students with a low-cost, high-value, study-away, service-learning program.

Presidio County in perspective

Presidio County’s population of 7,156, is 81.8% Hispanic, 14.8% White, and 3.4% Asian [13]. In Presidio County, 86.4% of the residents speak a non-English language, such as Spanish, Korean, and Tagalog. The Big Bend Regional Medical Center at Alpine, in nearby Brewster County, is the major provider of primary rural healthcare covering 12,000 square miles. The distance to this single hospital can range from 30 minutes to three hours away. A major contributing factor to health disparities for this region is the distance to the hospital. Furthermore, the limited number of ambulances and life flight helicopters exacerbates the issue of transportation and access to appropriate healthcare. Presidio County, a frontier region, has a 1:43 primary care clinician to patient ratio, with patients aged six to 17 years most likely to have healthcare coverage. The most distant town is Candelaria. It is a border village in Presidio County with about 70 inhabitants, no mayor or leading authority, and only a single Catholic church next to an abandoned two-room schoolhouse. Historically, there has been fluid interaction and passage between Candelaria and the Mexican town of San Antonio del Bravo across the Rio Grande River, but minimal documentation exists since this is a small, economically insignificant town that also lacks a border wall [14]. Nevertheless, this town is still in the US. Since the rudimentary infrastructure is so contrary to the typical metropolitan American image, this is a perfect example of a community that lacks resources that contribute to health disparities within our borders.

Based on the US Department of Agriculture (USDA) Economic Research Service’s data, approximately 42.80% of the population in Presidio County is considered low income with low access to a store for grocery shopping [15]. Of particular note, 62.69% of the Hispanic population has limited access to a store, a 20% increase compared to the entire county population. In 2014, there was one grocery store in Presidio County and six convenience stores. Besides these barriers to store-purchased foods or commonly found food desserts, citizens also experience limited ability for growing crops. In fact, the annual rainfall in the Presidio area is approximately 27.25 cm, making the Presidio fall under the classification of a desert environment [16-17].

These factors are some of the social determinants of health and they highlight a need in the community that goes beyond healthcare. It includes a need for effective education on how to eat a healthy, well-balanced diet despite the difficulty of accessing healthy food. It encompasses the difficulty in accessing reliable transportation to travel the sometimes vast distances to go to annual visits and undergo other preventative measures. The various limitations in this community affect their overall health in a different way compared to larger, more metropolitan areas.

Our overall goal is to expose students to health disparities to increase cultural competency by providing rich clinical field experiences in essentially ‘foreign’ environments found in our own backyard. We evaluated the effectiveness of this program to achieve the goals of exposure to health disparities and an increase in cultural competence and to measure the impact the study-away experience had on students’ future career goals. We qualify these based on the change in student’s professional trajectory, their mentality, and their awareness of health disparities after the study-away, service-learning trip.

## Materials and methods

Students from all health professions schools at the UTMB in Galveston, TX (medicine, physician assistant, nursing, occupational therapy, and physical therapy) were recruited through the Frontera de Salud program to join a mission trip to southwest Texas for a total of 45 students involved in the trips. Each mission trip lasted one week and allowed students to provide healthcare in the following underserved frontier communities in Presidio County: Alpine, Presidio, Marfa, and Candelaria. The students were chosen so that roughly four had at least moderate Spanish proficiency, that various health professional programs were represented, and that various levels of clinical skills would be present. Students were assigned to clinics in the area such that there were three to four students per clinician. Institutional Review Board approval was obtained from the UTMB (IRB #16-7894). 

Immediately after the trip, students who had participated were sent a follow-up survey via Google Forms to voluntarily fill out. This survey was adapted from a previous study that aimed to understand how students benefit from community outreach that integrates critical post-trip evaluation to create lasting changes in the students’ outlook on healthcare in underserved areas [8]. See Table 1 for the specific questions asked. The post-trip survey and survey responses were kept on a secured Google Drive for data management. The survey responses were downloaded into an Excel spreadsheet and each open-ended question was evaluated for recurring themes throughout the student responses (Table 2). Simple statistical analyses were performed for the close-ended questions.

**Table 1:** Open-ended Questions in the Survey

Open-Ended Questions
1. Why did you decide to participate in the Frontera de Salud Mission trip?
2. What were your key takeaways from this mission trip?
3. What did you expect it to be like once you were there?
4. What have you learned about yourself as a result of participating in this mission trip?
5. Have you come to see anything differently about the United States as a result of this trip?
6. Have you come to think differently about anything as a result of this mission trip?
7. What event during this trip has made the greatest impression on you?
8. What do you think might be the impact of this experience on you or on the community where you volunteered?

**Table 2:** Thematic Answers to Open-ended Questions in the Survey

Question:	Consolidated Themes	Number of Responses
What did you expect it to be like once you were there?	Surprised by the degree of health disparity/level of underserved	11
	Expecting the worst in regards to the quality and accessibility of healthcare	10
What have you learned about yourself as a result of participating in this mission trip?	Were glad to know that their knowledge of the Spanish language came into use	6
	The trip raised their desire to work with underserved populations	16
Have you come to see anything differently about the United States as a result of this trip?	Understand disparity better (health resources, accessibility)	15
	Recognize that “places like this exist in the US” referring to Candelaria and Presidio	10
What event during this trip has made the greatest impression on you?	Mobile Day Clinic: Candelaria, TX	21
	The appreciativeness of the community people for the students’ visit and appreciation of the local populations’ overall life struggles	2
	Learning about what rural medicine specifically entails	3
What do you think might be the impact of this experience on you or on the community where you volunteered?	Specific impact on community stated	3
	Recognition of health disparities	12
	Belief/hope that positive impact was made	15
	Motivated to incorporate underserved populations and/or rural medicine in their future career choices	13

Literature

With the assistance of librarians, existing literature on study-away programs was found using the Ovid database.

## Results

From 2016 to 2018, three mission trips to Presidio County, TX were completed. One mission was focused on community health and two were focused on medical preventative care. Table 3 provides a demographic summary of the trip participants who elected to complete the post-trip survey. There was a 75% response rate (36/45) for the three trips in total. The ages of the participants ranged from 21 - 31 years. Table 1 listed the questions asked on the post-trip questionnaire. Table 2 presented the major themes and the frequency at which they were mentioned for each question. 

**Table 3:** Survey Demographics MD: medical doctor; OT: occupational therapy; PT: physical therapy; RN: registered nurse

Sex	Number of Responses	%
Male	13	36.1
Female	23	63.9
Total	36	
Professional Program		
School of Medicine (MD)	27	75.0
Physician Assistant Studies (PA)	0	0.0
School of Nursing (RN)	6	16.7
School of Health Professions (OT, PT)	3	8.3
Race/Ethnicity		
Non-Hispanic White	14	38.9
Hispanic	13	36.1
Asian	9	25.0
African American	0	0.0

When asked why they chose to participate in the Frontera de Salud trip, 16 students responded that they wanted to experience medical work in an underserved community. A few mentioned that this experience was more manageable with their finances and schedule than other opportunities. For example, one student commented, “doing community service and being able to treat and take care of patients outside of the hospital are one of the key reasons I decided to go into (nursing). I have wanted to partake in many of the mission trips that UTMB has offered, but money and scheduling normally get in the way. This seemed like an amazing opportunity when I got the email, and I knew that this was something that I should take part (in).” When asked what they expected from the trip, 10 students replied that they were surprised by the level of disparity witnessed on the trip. One student replied, “While I knew Presidio and the surrounding area was medically underserved, I underestimated just how underserved they are.”

When asked what they learned about themselves as a result of this trip, 16 students responded that they discovered a need within themselves to work in some capacity with the underserved in the future. One student replied, “I have learned that I have a passion for serving the underserved and wish to continue helping those without access to care.” When asked if they see the US differently after this experience, 10 students noted they developed a better understanding of the level of disparity within the US. One student stated, “I knew there was a disparity in medical service in the US, but I was shocked to see it in person. It seemed like I was across the border in a third world country.” When asked to comment on the event that left the greatest impression on them, 21 responses were related to their experience in the frontier town of Candelaria. One student responded, “Our day trip to Candelaria was the most impactful for me. The fact that those people receive all of their health care once a week in the back rooms of a tiny church was difficult for me to swallow.” When asked about their perception of the impact of this experience on themselves or the community they helped, 15 responses were related to the theme of believing or hoping that a positive impact was made. One student replied, “I hope that we positively impacted the community we served during this trip. I really do want to provide for underserved populations in the future as a result of this trip.” Another common response to the personal effects of the trip was the desire to learn or improve their Spanish language fluency to better communicate with a large portion of the state's population. One participant stated, “I definitely need to learn Spanish. I felt very limited when trying to speak to patients whose only language was Spanish.”

## Discussion

Like other programs centered on service-learning within study-away experiences, the results from our reflective follow-up from the trips to southwest Texas suggest that this experience helps students become more receptive to the needs of diverse patient populations and aware of the health disparities in their own country. This heightened awareness should have a positive effect on their future work as healthcare professionals by influencing how they diagnose, treat, and follow-up with patients from different cultures and backgrounds. Current studies have found that physicians lacking resources and training in cultural competency report feeling unprepared when providing care to culturally and socially diverse patients [18]. Because this program provides exposure to working with an unfamiliar community before students become practitioners, it allows both time and opportunity to foster a sense of preparedness in treating a diverse patient population and to raise awareness of the contributing factors that lead to health disparities.

There are multiple discussions regarding the various physician training techniques used to improve cultural competencies, levels of empathy, and overall understanding of patients of different backgrounds. Current literature addresses student exposure to various types of community service learning outside the framework of clinical placements to influence their development of cultural humility, empathy, and a sense of social justice [19]. Our survey addresses the immediate impact of such trips on the students involved, inviting introspection and societal critiques from the students. Individual impacts will stay with the students and guide their future interaction with patients no matter how they differ from each other. At the community level, students retain their awareness of health disparities and social inequities seen here in Texas, their own backyard as it were, as 90% of our students are Texas residents. By encouraging reflection via the post-test survey immediately afterward, each student must recall all that was seen and done, thereby reinforcing the neural connections that house those memories. This quick turn-around survey captures the immediate impact of these trips on the student’s mindset while evaluating the potential effect of these experiences on cultural competency and health disparity awareness. There is a need in medicine to continually promote the development of empathy at the level of medical education, and hands-on experiences have been found to promote this development [20]. Empathy gained through study-away programs such as ours, enhance the compassion and understanding of future physicians and other healthcare professionals and potentially instill in them the desire to advocate for underserved patient populations. In areas such as South Texas, there is a need for culturally sensitive physicians, particularly for the increasing Spanish-speaking population. Through service-learning opportunities such as this, students can begin to adapt to diverse healthcare environments, overcoming cultural barriers, navigating the different language and communication needs, be it by tele-translating or through the provider’s second language, and become comfortable with the uncomfortable.

That the most impoverished city was the most impactful event speaks for itself. The experience of practicing medicine in a church with the patients waiting the entire day to see the one doctor in curtain drawn rooms left a clear impact on many students involved. The struggles that students experienced in the frontier town, as well as the less rural hospital, demonstrate that being rural/frontier changes how care is provided. Students arrive at their own broader understanding of social inequities and health disparities and are better able to extrapolate the range of health disparities contained within the United States’ borders and better able to define their future role as healthcare providers and community advocates.

## Conclusions

These service-learning, study-away opportunities not only affect students’ outlook on future ways of practicing and interacting with patients but also introduce or augment their desire to work with an underserved population. For some students, these trips have created a desire to work in a rural area to improve healthcare in a community with currently inadequate care. In order to continue observing the impact this type of learning has on healthcare profession students, it is essential to encourage reflection and a critical consciousness after each trip. Gaining insight into the role of economic and political factors in health disparities allows the student to consider the patient's socioeconomic background when determining treatment options. In the future, we will strive to make the trip increasingly more interprofessional to further develop professional skills gained on the trip. With feedback opportunities, students can also help improve the programs to maximize the benefit to both the community and students. We recommend that other study-away (and for that matter, study-abroad) programs capture student impressions of the experience quickly after returning home. Assessment of student impressions can confirm or deny that the purpose of each program is being achieved and reinforce the experiences with the student. Student-led programs, in particular, can utilize student and community feedback to quickly inform the program if the value is being received by the students and the community and adjust accordingly. If study-away programs with less financial burdens can achieve the same professional development goals as the more expensive study-abroad programs, increased effort must be given to developing programs such as this one.
